# Dynamically Rough Boundary Scattering Effect on a Propagating Continuous Acoustical Wave in a Circular Pipe with Flow

**DOI:** 10.3390/s18041098

**Published:** 2018-04-05

**Authors:** Anna V. Romanova, Kirill V. Horoshenkov, Anton Krynkin

**Affiliations:** 1 Faculty of Engineering & Science, University of Greenwich, Central Avenue, Chatham, Kent ME4 4TB, UK; 2 Department of Mechanical Engineering, University of Sheffield, Mappin Steet, Sheffield, South Yorkshire S1 3JD, UK; k.horoshenkov@sheffield.ac.uk (K.V.H.); a.krynkin@sheffield.ac.uk (A.K.)

**Keywords:** boundary scattering, dynamic roughness, sine wave, pipe flow, pipe acoustics

## Abstract

The pattern of the free surface of the turbulent flow in a partially filled circular pipe contains information on the underlying hydraulic processes. However, the roughness of the free surface of flow and its temporal variation in a pipe is a dynamic and non-stationary process that is difficult to measure directly. This work examines a new acoustic method that is used to study the characteristics of the free surface roughness under controlled laboratory conditions. The acoustic method makes use of a continuous sine wave that is transmitted through the air above the turbulent flow of water over a section of the pipe instrumented with an array of wave probes and microphones. The results obtained for a representative range of flow regimes and variety of pipe bed conditions illustrate that it is possible to unambiguously relate variations in the recorded acoustic field to the standard deviation in the free surface roughness and mean flow depth. These variations are clearly linked to the hydraulic friction factor of the pipe, which is shown to be related to airborne acoustic data obtained non-invasively.

## 1. Introduction

There is a general lack of evidence on the relationship between the airborne acoustic field in a partially filled pipe and the behaviour of the free water surface of the hydraulic flow in the presence of discrete bed roughness. Theories exist for sound propagation in the presence of a rough boundary [1,2,3,4,5], but not for a partially filled round pipe in which the flow surface is dynamically rough. To the best of the authors’ knowledge, there has not been any experimental work on sound propagation in a partially filled pipe with a dynamically rough flow surface. For example, Ref. [1] is largely relevant to sea surface scattering; Ref. [2] is relevant to sound propagation in a circular waveguide with static roughness; Ref. [3,4,5] are relevant to outdoor sound propagation in the presence of a rough impedance ground. 

The concept presented in this paper is to relate the statistical characteristics of the dynamically rough water surface to the statistical characteristics of the scattered acoustical signal. A simple theoretical way to account for the flow surface roughness in a partly filled pipe is to introduce the notion of the eigenvalue correction ($\xi_{0,2}$) to the acoustic wavenumber ($k=\omega_{0}/c$) in the airborne section of the pipe affected by the roughness of the dynamic surface, ($\omega_{0}$) being the angular frequency and (c) is the sound speed in air. We consider the case, when: (i) the acoustic wavelength (λ) is sufficiently large compared to the diameter of the pipe ($d=2R_{c}$, $R_{c}$ being the pipe radius); and (ii) the mean roughness height ($\sigma_{h}$) is much smaller than the acoustic wavelength. In this case, it is possible to limit sound propagation in the pipe to one plane wave only whose acoustic wavenumber can be approximated with the following expression [6]:

(1) $$k_{00}=k+\xi_{0,2},$$

where the eigenvalue correction ($\xi_{0,2}$) to the acoustic wave number in the air (k) is determined as the average over a representatively large number of surface roughness realizations or period of time. It was shown in [6] that the value of this correction can be expressed as follows:

(2) $$\xi_{0,2}=\frac{i\sigma_{h}^{2}}{2E_{h}^{2}}k^{2}W(2k).$$

In Equations (1) and (2) $i=\sqrt{-1}$, W(2k) is the Fourier spectrum of the spatial correlation function for the instantaneous surface elevation and $E_{h}^{}$ is the mean water level in the pipe. 

The above relations suggest that the eigenvalue correction to the acoustic wave number for a given frequency of sound is purely imaginary. It is proportional to the square of the mean roughness height and the square of the frequency of sound because of the presence of the terms $\sigma_{h}^{2}$ and $k^{2}$ in Equation (2). The spatial correlation function also influences the behaviour of Equation (2). In the case when $l/E_{h}<<1$ or when the correlation radius in the surface roughness (l) is relatively independent of the flow regime, its effect on the eigenvalue correction is relatively small. 

Let us assume that a sinusoidal signal $s\left(t\right)=a_{0}e^{-i\omega_{0}t}$ is used to excite the fundamental mode in a pipe with a dynamically rough surface caused by the turbulence in the hydraulic flow. If the hydraulic process is not stationary, resulting in the slow fluctuation of the mean water height over function of time (i.e., the characteristic time period of change in the value of $\sigma_{h}$ is much longer than the period in the harmonic sound wave ($2\pi /\omega_{0}$)), then the eigenvalue correction ($\xi_{0,2}$) will also fluctuate in proportion to the squared mean roughness height ($\sigma_{h}$). In this case, the time-dependent sound pressure at the receiver will be amplitude-modulated, i.e., $p\left(t\right)=\left(p_{0}+p^{\prime }\left(t\right)\right)e^{-i\omega_{0}t+ikx}$, where $p_{0}$ is the sound pressure in the absence of roughness and $p^{\prime }\left(t\right)$ is the perturbation term that relates to the change in the mean roughness height in the dynamically rough free-water surface and varies much more slowly than the exponential term $e^{-i\omega_{0}t+ikx}$. At the receiver position, the amplitude of the sound pressure will be affected by the roughness so that it is average over a number of rough surface realizations. If the dynamic roughness is an erratic process, then it is root mean square value averaged over a time interval can be expressed as:

(3) $$<p>≅p_{0}\left(1+\frac{\sigma_{A}^{2}}{2p_{0}^{2}}\right),$$

where $\sigma_{A}$ is the standard deviation in the acoustic pressure amplitude. Because the ratio $\frac{\sigma_{A}^{2}}{p_{0}^{2}}\ll 1$, Equation (3) makes use the Taylor series expansion of the square root function $\sqrt{1+x^{2}}\approx 1+\frac{x^{2}}{2}$ to reduce its complexity. The theoretical analysis of the sound field in a waveguide with a rough wall (Equation (53) in [6]) suggests that the mean sound pressure amplitude is $<p>=p_{0}e^{i(k+\xi_{0,2})x}$. If the mean roughness height is small, i.e., $d>>\sigma_{h}$, then $e^{i\xi_{0,2}x}≅1-\frac{\sigma_{h}^{2}}{2d^{2}}k^{2}W(2k)x$ so that:

(4) $$\frac{\sigma_{A}^{2}}{p_{0}^{2}}∼-\frac{\sigma_{h}^{2}}{d^{2}}k^{2}W(2k)x.$$

The authors note that the waveguide width used in the 1D case considered in [6] is replaced here with the mean water level ($E_{h}$), which is valid assuming that the wave propagating in the pipe is plane so that the problem can be effectively reduced to 1D. Equation (4) suggests that the mean roughness height and standard deviation in the sound pressure amplitude are proportional, i.e., $\sigma_{A}∼\sigma_{h}$.

The main purpose of this work is therefore to illustrate experimentally that the amplitude variations described by Equation (4) can be detected and related unambiguously to the mean roughness height variations that are measured independently with an array of wave probes. In this way, the free surface roughness of the flow in a partially filled pipe can be measured non-invasively and linked to the hydraulic processes. 

## 2. Materials and Methods 

**Figure 1:**
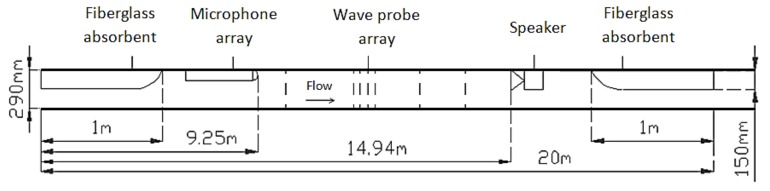
Pipe arrangement with presence of wave probe and microphone arrays, speaker, and fiberglass absorbents. Not to scale.

The experiments were carried out in the transparent (Perspex) circular pipe with the internal diameter of 290 mm (d) and 20 m in length, as in Figure 1. 

The pipe was installed on a flat, solid, steel beam at a slope of 1 in 2000. In the experiments, the water discharge and position of the gate at the downstream end of the pipe were controlled to achieve a range of flow levels and velocities for a uniform flow regime. The discharge was controlled by a mechanical valve and calibrated using a water weighting tank.

**Figure 2:**
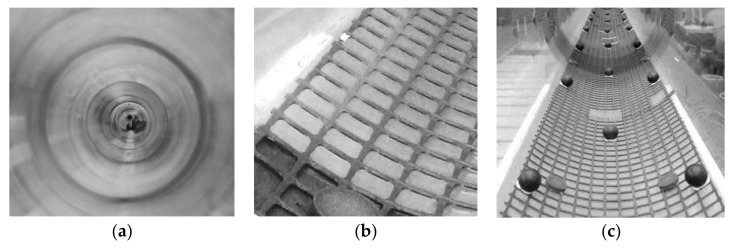
Three pipe bed conditions: clean/empty pipe (**a**); pipe with mesh (**b**) and pipe with 8D separated spheres (**c**).

The pipe bed was artificially roughened to influence the water surface pattern and the resultant mean roughness height of the free water surface. In total, eight pipe bed conditions were studied. Examples of these conditions are illustrated in Figure 2. For the clean pipe condition (condition C) the pipe wall material roughness was estimated to be 0.01 mm, Figure 2a. In another experiment, a 20 m long, 200 mm wide square mesh, with the mesh grid being 2 mm thick, 4 mm wide and grid square 12 mm long (condition M) was attached to the bottom of the pipe. Small powerful magnets were used to force the mesh to fit the pipe bed curvature as shown in Figure 2b. This mesh was completely submerged in water for all the flow regimes studied in this experiment. For the other six roughness conditions, the same mesh was roughened with 25 mm diameter (D) spheres that were arranged in a hexagonal pattern as shown in Figure 2c. The separation between the spheres was fixed along the width of the mesh, but varied in the streamwise direction. The streamwise separation between the spheres was set to 4D, 6D, 8D, 10D, 12D and 16D in the six different experiments.

To capture the behaviour of the free surface roughness, an array of seven resistance wave probes was used. The wave probes were located in the middle of the pipe between 9 m and 16 m downstream. The exact locations of these wave probes are quoted in Table 1, where the distance is measured from the pipe inlet. This part of the pipe was chosen for the water roughness measurements because the turbulent water flow in this section was fully developed, steady and uniform. The turbulent condition of the pipe flow was ensured through a relatively high Reynolds number, $Re=\left(\rho E_{h}V\right)/\eta$ which was in the range of 10,000–34,000 for the experimental conditions presented in Table 2. Here $E_{h}$ is the mean flow depth obtained as a time average from all seven wave probes and V is the mean flow velocity, η and ρ are the dynamic viscosity and density of water taken at 15 °C. 

The wave probe signals were received on the wave probe monitor [7] and digitized with a National Instrument (NI) DAQ, Type USB-6356 (Hungary) [8] connected to a NI Control Unit, Type PIXe-1082 [9]. The LabView software was used to acquire the wave probe data and to control the acoustic data collection process. The data were acquired at a sampling frequency of 22,100 Hz in 20 s long packets over a time interval of 200 s. These data were saved in 10 separate files that were then analysed collectively. The time interval between the 10 continuously recorded files was approximately 3 s; this time was required for the acquisition system to save the data on the hard disk.

**Figure 3:**
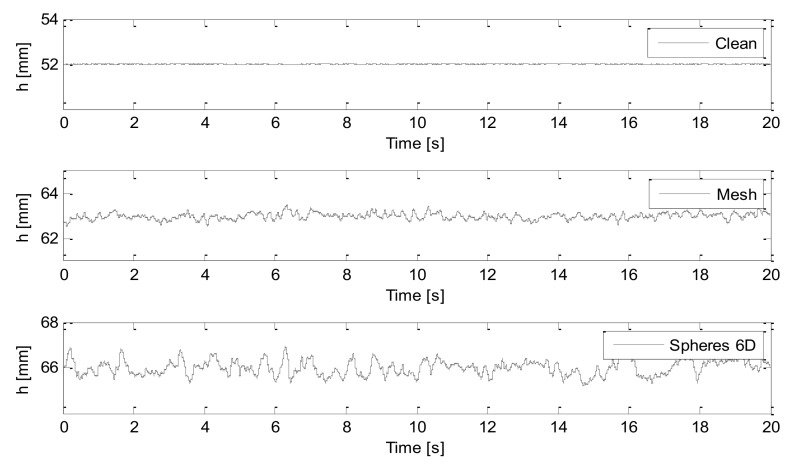
Examples of the water level fluctuations recorded on wave probe 1 (h) measured for 20 s in the clean pipe at *V* = 0.24 m/s and h = 52 mm, pipe with the mesh at *V* = 0.21 m/s and h = 63 mm, and pipe with 6D spheres arrangement at *V* = 0.20 m/s and h = 66 mm.

The mean water level was calculated from the seven sets of wave probe data from

(5) $$E_{h}=\frac{1}{7T}\sum_{j=1}^{7}\int_{0}^{T}h_{j}^{}(t)dt,$$

where $h_{j}$ are the water level data recorded on the probe (j) over the time interval of T=20 s. Figure 3 shows examples of the water levels recorded on wave probe 1 for three hydraulic regimes with similar mean flow velocity, but a progressively increased wall roughness.

The mean free surface roughness height was calculated from

(6) $$\sigma_{h}=\frac{1}{7}\sum_{j=1}^{7}\sqrt{\int_{0}^{T}{\left[h_{j}^{}(t)-{\bar{h}}_{j}\right]}^{2}dt},$$

where $\bar{h_{j}}$ is the mean value of $h_{j}\left(t\right)$ over the time interval of T=20 s.

The Darcy–Weisbach friction factor (f) for a circular pipe is calculated as

(7) f=8gResfV2,

where g is specific gravity, $s_{f}$ is the pipe bed slope, V is the mean flow velocity and Re is the Reynolds number. 

**Figure 4:**
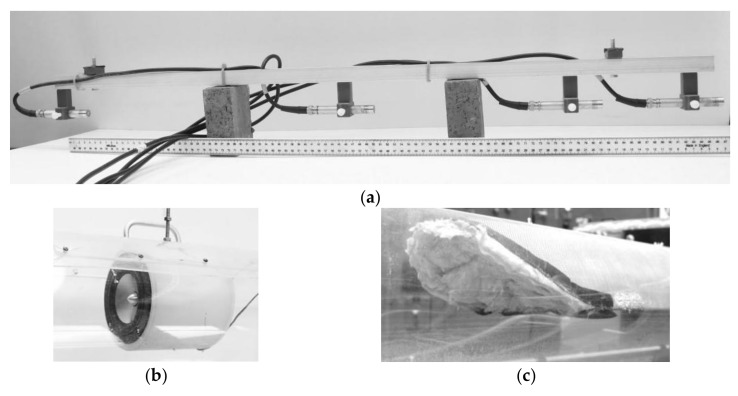
Microphone on a steel bracket (**a**); speaker attached to the pipe top (**b**); sound absorbent occupying top half of pipes cross section at pipe outlet (**c**).

An array of four measuring microphones, Bruel and Kjaer Type 4190-C-001 (Denmark) [10] was installed at 9.25 m from the inlet of the pipe as shown in Figure 4a. The microphones were calibrated using a standard 94 dB calibrator, Bruel and Kjaer Type 4231 (Denmark) [11] so that their sensitivity mismatch was compensated. A hi-fi, medium range speaker, Visaton TI100 (China) [12] was located at 14.94 m and oriented towards the microphone array, Figure 4b. To reduce the strength of any acoustic reflections from the pipe end and outside incidental noise, sound absorbers were placed at both open ends of the pipe, Figure 4c. The acoustic signal was a 500 Hz pure tone emitted and recorded continuously for 200 s using the same data acquisition equipment as described in the previous section. This frequency was adopted because: (i) it is below the frequency of the first cross-sectional mode of the pipe (665 Hz); (ii) the acoustic wavelength at this frequency is small enough for the dynamic free surface roughness to have an effect on the sound pressure in the pipe; (iii) at this frequency, the sound absorbing terminations, Figure 4c, are able to absorb over 95% of the incident sound energy. 

The wave probe and acoustic data collection was synchronized to run simultaneously. The acoustic signals used in this analysis were filtered using a 3rd order 400–600 Hz Butterworth bandpass filter. In this frequency range, the signal to noise ratio for the 500 Hz signal was greater than 66.4 dB for all the experiments. 

The envelope (amplitude) of the acoustic pressure, $p_{j}\left(t\right),$ recorded on each of the four microphones, j=1,2,3,4, was extracted from

(8) $$p_{0,j}+p_{j}^{\prime }(t)=\left|{\hat{p}}_{j}(t)\right|,$$

where ${\hat{p}}_{j}(t)=p_{j}(t)+i{\hat{p}}_{j}(t)$ and ${\hat{p}}_{j}\left(t\right)$ is the Hilbert transform of the signal, $p_{j}\left(t\right)$, and $p_{0,j}$ is the mean sound pressure. If the acoustic pressure $p_{j}\left(t\right)$ is a harmonic process, i.e., $p_{j}\left(t\right)=A_{j}\left(t\right)\cos\omega t$, where A(t) is the slowly varying amplitude, then its Hilbert transform is ${\hat{p}}_{j}\left(t\right)=A\left(t\right)\sin\omega t$ so that ${\hat{p}}_{j}(t)=A_{j}(t)e^{i\omega t}$ and $\left|{\hat{p}}_{j}(t)\right|=A_{j}(t).$ Once the envelope was extracted, the mean standard deviation in the acoustic signal envelope for all four microphones was calculated from

(9) $$\sigma_{A}=\frac{1}{4}\sum_{j=1}^{4}\sqrt{\frac{1}{T}\int_{0}^{T}{\left[p_{j}^{\prime }(t)\right]}^{2}dt}.$$

The mean amplitude of the continuous sound pressure wave envelope, measured with the four microphones, which is dimensionless as is referred to 1 V, was calculated from

(10) $$E_{A}=\frac{1}{4}\sum_{j=1}^{4}\sqrt{\frac{1}{T}\int_{0}^{T}p_{0,j}^{}(t)dt}.$$

**Figure 5:**
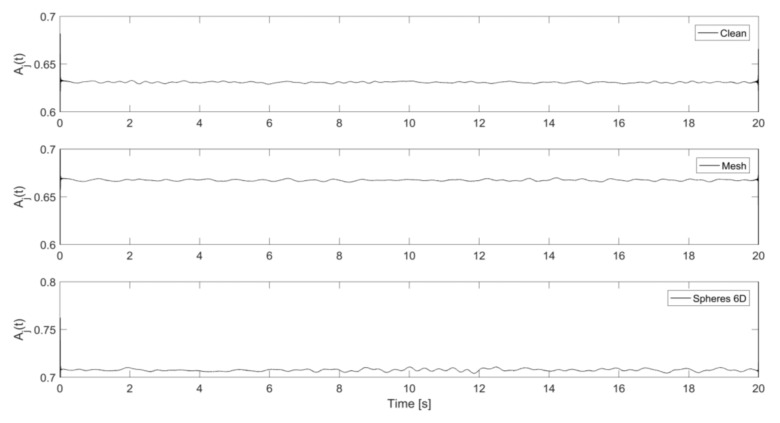
Examples of the continuous wave envelope recorded on microphone 1 for 20 s in the clean pipe at V = 0.24 m/s and h = 52 mm (**top**); pipe with the mesh at V = 0.21 m/s and h = 63 mm (**middle**); and pipe with 6D spheres arrangement at V = 0.20 m/s and h = 66 mm (**bottom**).

Figure 5 presents examples of the measured, time-dependent acoustic pressure envelopes, $\left|{\hat{p}}_{j}(t)\right|=A_{j}(t)$, for three hydraulic regimes where the wall roughness was progressively increased.

The probability density function of the envelope data $\left|{\hat{p}}_{j}(t)\right|$ was also calculated as the probability of $P(x\leq \left|{\hat{p}}_{j}(t)\right|\leq x+\Delta x)$ for a given amplitude limit of x and amplitude bandwidth Δx and fitted with the Gaussian distribution function, $P(x)=\frac{1}{\sqrt{2\pi }\sigma_{A}}e^{-\frac{{\left(\left|{\hat{p}}_{j}(t)\right|-E_{A}\right)}^{2}}{2\sigma_{A}^{2}}}$, to prove that the signals obey closely the normal statistical distribution. 

## 3. Results

**Figure 6:**
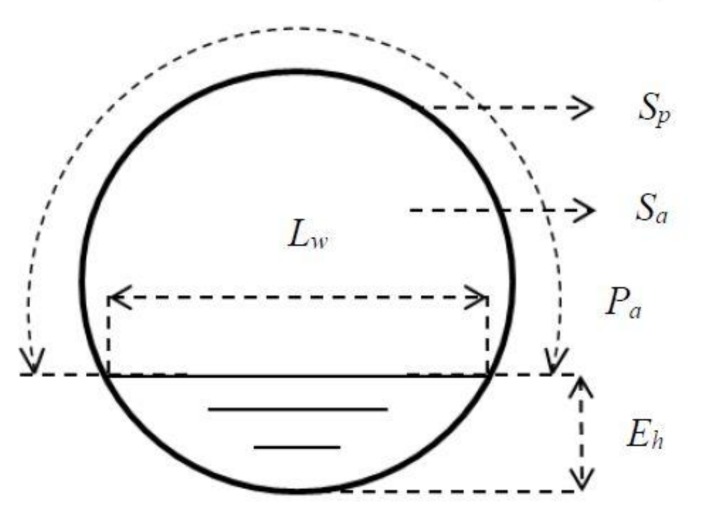
Geometric parameters of a cross section of the pipe with the presence of flow.

Table 2 presents the hydraulic and acoustic characteristics for all experimental regimes considered. The first column in this table shows the flow regime number. The second column shows the acronym used to denote the roughness condition. The following columns in Table 2 show the mean flow depth obtained through the wave probes ($E_{h}$), mean flow velocity (V), Reynolds number (Re), Darcy–Weisbach friction factor (f), the hydraulic parameters ratios ($L_{w}/P_{a}$) and ($S_{a}/S_{p}$). Here $L_{w}$ is the flow free surface width measured across the pipe, $P_{a}$ is the perimeter of the dry section of the pipe, $S_{a}$ is the dry cross-sectional area of the pipe (air) and $S_{p}$ is the cross section of the whole pipe. Figure 6 demonstrates the free surface width and other geometric parameters of the pipe. Column 9 in Table 2 presents the mean free surface roughness height ($\sigma_{h}$), obtained from all seven wave probes for the whole of the recorded sample length (T=200 s). Column 10 presents the mean free surface roughness height normalized using the pipe cross-sectional geometry parameter, $\sigma_{h}^{*}=\sigma_{h}L_{w}/P_{a}$. Furthermore, Table 2 presents the acoustic characteristics, where columns 11–13 show the mean amplitude of the continuous sound pressure wave envelope ($E_{A}$), the standard deviation in the continuous sine wave envelope ($\sigma_{A}$) and its normalized value, $\;\sigma_{A}^{*}=\sigma_{A}S_{a}/S_{p}$.

There are three reasons for the above normalizations. First, to take into account the variations in the dry part of the pipe cross section and variations in the width of the free flow surface that are caused by the changes in the water level in the pipe in the reported experiments. Second, to take into the account the relative change in the sound pressure emitted by the speaker, which is caused by the change in the dry cross section of the pipe. Third, to account for the changes in the dry cross section of the pipe and the width of the rough flow surface so that their effect on the acoustic pressure fluctuations in the pipe can be normalized. The proposed normalization should enable us to generalize the results of these experiments to other pipes of different diameters with different wall roughness conditions and variable levels of flow.

**Figure 7:**
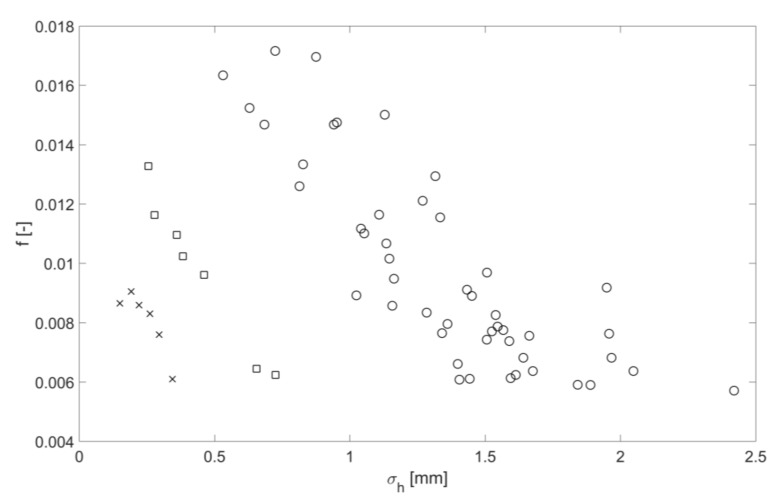
Friction factor as a function of mean free surface roughness height for three roughness types, clean pipe (cross), pipe with mesh (squares), pipe with spheres (circles).

**Figure 8:**
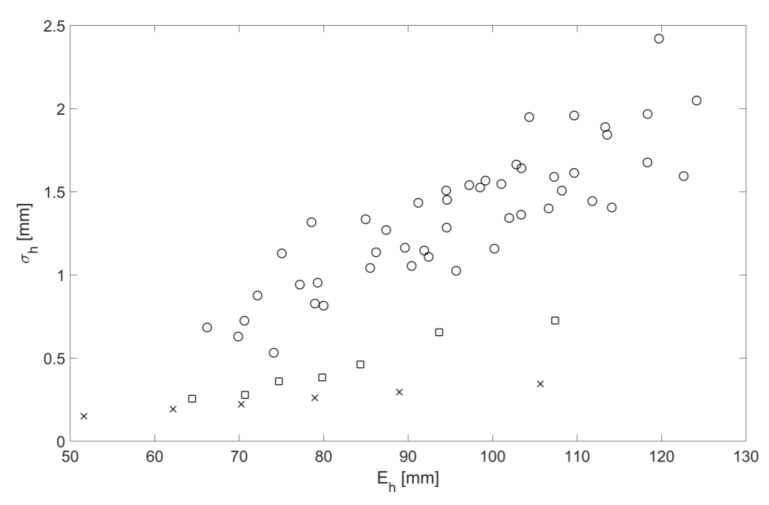
Mean free surface roughness height as a function of mean flow depth for three bed roughness types: clean pipe (cross), pipe with mesh (squares), pipe with spheres (circles).

**Figure 9:**
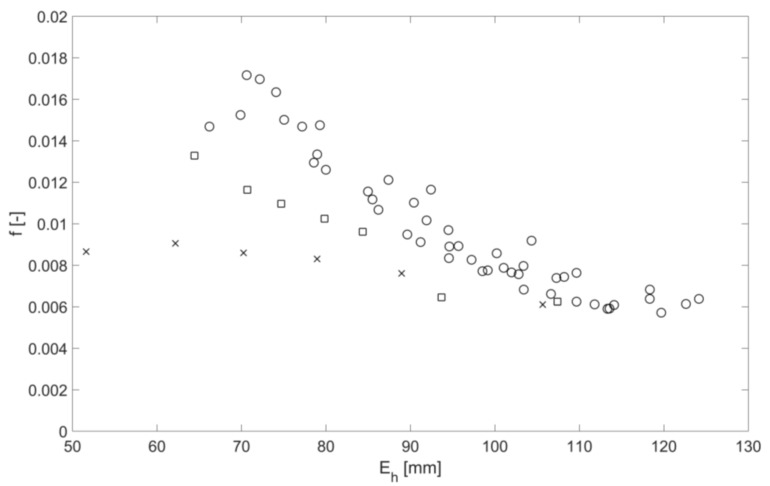
Friction factor as a function of the mean flow depth for three bed roughness types: clean pipe (cross), pipe with mesh (squares), pipe with spheres (circles).

For all the flow conditions listed in Table 2, Figure 7 shows the friction factor as a function of mean free surface roughness height. The data shown in this figure group in three sets for the clean pipe (crosses), pipe with the mesh (squares) and pipe with spheres (circles). Within each of these sets the dependence of the mean free surface roughness height on the friction factor is approximately linear. However, there is a considerable scatter in the data in the case of the pipe with the spheres. Figure 8 shows the dependence of the mean free surface roughness height on the mean flow depth for the same three roughness conditions. Similar to the result shown in Figure 7 the data group in three sets within which the dependence of the mean free surface roughness height on the mean flow depth is approximately linear with a considerable scatter in the data in the case of the pipe with the spheres. As expected, the mean free surface roughness height increases with the increase of the pipe bed roughness and mean flow depth. This can be associated with a higher mean flow velocity in deeper flows with a higher turbulence kinetic energy and larger turbulence scales [13] which are affected by a higher bed roughness. The results in Figure 9 show the relationship between the friction factor and mean flow depth. The friction factor decreases with the increased mean flow depth. This result is consistent with the findings reported by Yoon et al. in [14]. This means that the friction factor reduces with the increase in the mean free surface roughness height, which can effectively serve as a friction factor estimate. 

**Figure 10:**
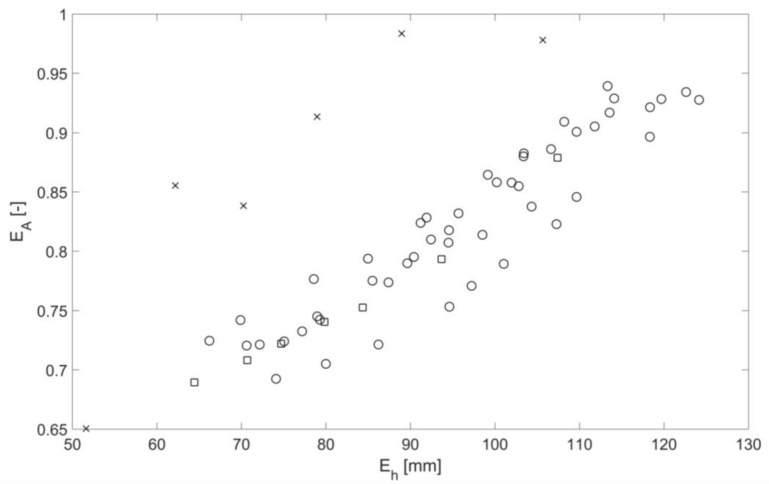
Continuous sound pressure wave envelope as a function of mean flow depth for three bed roughness types: clean pipe (cross), pipe with mesh (squares), pipe with spheres (circles).

Figure 10 demonstrates a relationship between the mean amplitude of the continuous sound pressure wave envelope and mean flow height. The dependence is close to linear for a given group of regimes. The general trend is that the mean amplitude of the continuous sound pressure wave envelope increases with the increased flow depth. The deviation from a general trend exists at low depths, particularly in the case of the pipe with mesh at the bed. This suggests that some normalization of the obtained data is required. 

It make sense that the change in the mean amplitude of the continuous sound pressure wave envelope ($E_{A}$) should be linked to the change in dry cross-sectional area of the pipe ($S_{a}$). Let us assume that the acoustic force acting on a volume of air in the pipe ($F_{s}$) is constant in all regimes because it is related to the speaker characteristics, which we kept constant for all the experiments. The amplitude of this force is

(11) $$F_{s}=S_{a,n}p_{n},$$

where $p_{n}$ is the measured acoustic pressure in the dry area of pipe and index *n* refers to the flow regime index (see Table 2). The dry cross-sectional area over which this force is applied is defined by the radius of pipe ($R_{c}$) and the mean water depth ($E_{h}$):

(12) $$S_{a}=\pi R_{c}^{2}\left\{1-\frac{1}{\pi }\left[\cos^{-1}\frac{R_{c}-E_{h}}{R_{c}}-\frac{1}{2}\sin\left(2\cos^{-1}\frac{R_{c}-E_{h}}{R_{c}}\right)\right]\right\}.$$

In the case when the mean water depth is relatively close to the radius of the pipe, i.e., $\frac{R_{c}-E_{h}}{R_{c}}\ll 1$, the cross-sectional area of the dry pipe can be simplified to

(13) $$S_{a}\approx \pi R_{c}^{2}\left[\frac{1}{2}+\frac{2}{\pi }\frac{R_{c}-E_{h}}{R_{c}}-\frac{1}{3\pi }{\left(\frac{R_{c}-E_{h}}{R_{c}}\right)}^{3}\right]=\pi R_{c}^{2}\left(\frac{1}{2}+\frac{5}{3\pi }\right)-E_{h}R_{c}\left(1+\frac{E_{h}}{R_{c}}-\frac{E_{h}^{2}}{3R_{c}^{2}}\right).$$

By relating the change in sound pressure with the first flow regime, Newton’s second law, shown in Equation (11), can be employed to derive

(14) $$\frac{E_{A,n}}{E_{A,1}}=\frac{S_{a,1}}{S_{a,n}},$$

where the mean amplitude of the wave envelope is related to the acoustic pressure as follows:

(15) $$E_{A,n}=<\left|p_{n}\right|>.$$

**Figure 11:**
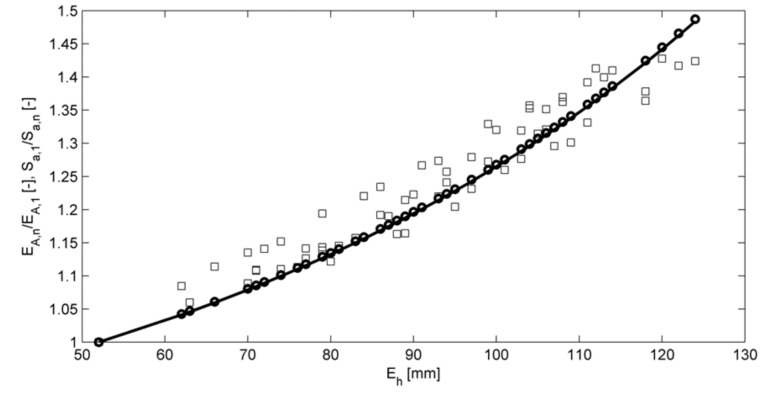
Normalized mean amplitude of the continuous sound pressure wave envelope (squares) plotted against the mean flow depth. The solid line represents Equation (12) and circles represent Equation (13), for all conditions, excluding clean pipe.

Figure 11 demonstrates the relationship between acoustic pressure and cross-sectional area derived in Equation (14) for all flow regimes except clean pipe. It is observed that any increase in wave envelopes depends on the water level as a cubic polynomial consistent, with the approximated cross-sectional area obtained via Equation (13).

**Figure 12:**
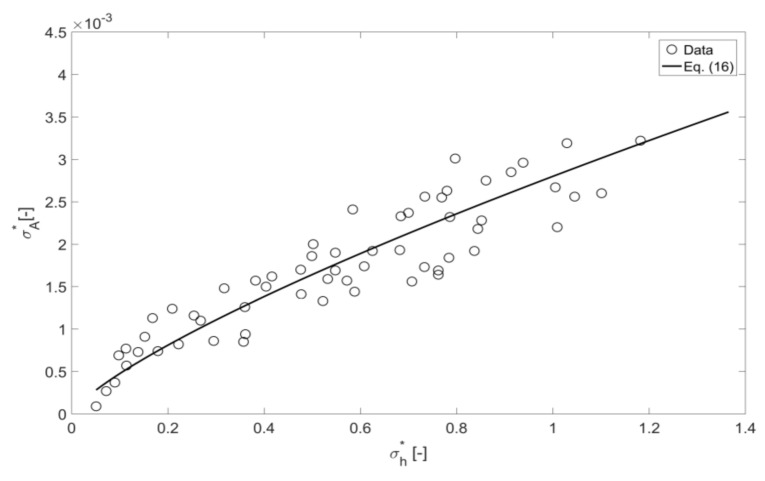
Normalized standard deviation in the continuous sine wave envelope as a function of the normalized mean free surface roughness height for all conditions, with *R*^2^ = 0.85, Equation (16).

Figure 12 shows the relation between the normalised standard deviation in the continuous sound pressure wave envelope and the normalised standard deviation in mean free surface roughness height plotted for all the conditions listed in Table 2. The dependence is not far from linear, which confirms the assumption [6] that the sound pressure fluctuations are likely to be directly proportional to the mean free surface roughness height. Because of the change in the mean flow depth in the pipe it makes sense to apply normalization to make this result non-dimensional and applicable to pipes of arbitrary diameter. For this purpose we study the dependence between the normalized quantities of $\sigma_{A\;}^{*}$ and $\sigma_{h}^{*}$, which are defined in Table 2. The following empirical relation between the normalized standard deviation in the acoustic wave envelope and the normalized free surface roughness height standard deviation can be proposed by

(16) $$\sigma_{A}^{*}={\left(0.0028\;\sigma_{h}^{*}\right)}^{0.77},$$

which exhibited a fit of *R*^2^ = 0.85, where *R*^2^ is the coefficient of determination. Similar to the results obtained in some other experimental studies where the pulse signal was used (e.g., [4,5,15]), the fluctuations in the acoustic wave envelope were found to increase with the increased fluctuations of the dynamic roughness of the water surface waves. 

## 4. Conclusions

New data on the behaviour of the free water surface and amplitude of the airborne sound wave transmitted above a flow of water have been obtained using an experimental rig consisted of a partially filled circular pipe equipped with wave probes and microphones. The microphones and a speaker have been installed inside the pipe, close to the top, and separated by a relatively long distance. The reported experiments have been conducted for a representative range of hydraulic and pipe bed conditions. 

It has been shown that the amplitude of the continuous airborne sound wave ($E_{A}$) relates directly to the mean water level ($E_{h}$) in the pipe. This relation has been derived theoretically and confirmed with data for all the flow conditions studied. Another relation has been observed between the amplitude of the continuous airborne sound wave ($E_{A}$) and the Darcy’s friction factor (*f*). This equation is more complex and it deserves further studies. A simple empirical equation (Equation (16)) has been proposed to link the normalized standard deviation in the amplitude of the continuous sound wave ($\sigma_{A}^{*}$) with the normalized standard deviation in the mean free water surface roughness height ($\sigma_{h}^{*}$). 

It has been found that the mean free water surface height depends strongly on the flow condition and increases with the increased bed roughness. The statistical behaviour in the sound pressure amplitude ($\left|\hat{p_{j}}\left(t\right)\right|$) and instantaneous water level ($h_{j}\left(t\right)$) is close to Gaussian with the means being $\left(E_{A}\right)$ and $\left(E_{h}\right)$, and standard deviations being $\left(\sigma_{A}\right)$ and ($\sigma_{h})$, respectively. 

These findings help to infer the hydraulic friction factor and mean flow velocity in a partially filled water pipe with a known bed slope from airborne acoustic data measured in the pipe above the flow. It has been shown that the normalized standard deviation in the continuous sine wave envelope can be related to the normalized mean free surface roughness height through Equation (16). The normalized mean amplitude of the continuous sound pressure wave envelope can be related to the mean flow depth through Equation (12). Figure 7 can be used to provide an estimate of the hydraulic friction factor from the mean free surface roughness height. The Darcy law (Equation (7)) can then be used to estimate the flow velocity for a given pipe slope, acoustically inferred hydraulic friction factor and mean flow depth. 

The proposed method can be attractive for non-invasive flow characterization in a partially filled pipe because it is relatively simple and it does not require any instrumentation to be submerged in the flow of water. Because of the normalization applied, these results should be generic and independent of the pipe diameter.

## Figures and Tables

**Table 1:** Equipment list and relevant locations in the pipe.

Equipment	Number and Position from the Pipe Inlet						
	1	2	3	4	5	6	7
Wave probes	9.60 m	11.55 m	11.62 m	11.67 m	11.70 m	12.80 m	13.80 m
Microphones	9.25 m	9.09 m	8.77 m	8.29 m	-	-	-
Sound absorbing termination	1 m	19 m	-	-	-	-	-
Speaker	14.94 m	-	-	-	-	-	-

**Table 2:** The summary of all experimental regimes with measured hydraulic characteristics.

1	2	3	4	5	6	7	8	9	10	11	12	13
No.	RC	$E_{h}$ [mm]	*V* [m/s]	*Re* [-]	f [-]	$L_{w}/P_{a}$ [-]	$S_{a}/S_{p}$ [-]	$\sigma_{h}$ [mm]	$\sigma_{h}^{*}$ [-]	$E_{A}$ [-]	$\sigma_{A}$ [-]	$\sigma_{A}^{*}$ [-]
1	C	51.62	0.378	10,431	0.00865	0.337	0.880	0.150	0.051	0.6504	0.00011	0.00009
2		62.18	0.402	13,093	0.00905	0.377	0.843	0.192	0.072	0.8554	0.00033	0.00027
3		70.24	0.435	15,768	0.00859	0.406	0.813	0.221	0.090	0.8384	0.00046	0.00037
4		78.96	0.465	18,629	0.00830	0.436	0.780	0.261	0.114	0.9135	0.00073	0.00057
5		88.96	0.511	22,598	0.00760	0.469	0.740	0.295	0.138	0.9835	0.00098	0.00073
6		105.65	0.610	30,965	0.00610	0.521	0.671	0.344	0.179	0.9781	0.00110	0.00074
7	M	64.43	0.337	11,328	0.01328	0.385	0.835	0.255	0.098	0.6894	0.00083	0.00069
8		70.69	0.375	13,664	0.01163	0.407	0.811	0.278	0.113	0.7081	0.00095	0.00077
9		74.71	0.395	15,114	0.01096	0.421	0.796	0.360	0.152	0.7221	0.00115	0.00091
10		79.83	0.421	17,011	0.01024	0.439	0.776	0.383	0.168	0.7404	0.00145	0.00113
11		84.36	0.444	18,818	0.00961	0.454	0.758	0.461	0.209	0.7526	0.00164	0.00124
12		93.69	0.566	26,122	0.00645	0.484	0.721	0.655	0.317	0.7933	0.00206	0.00148
13		107.40	0.607	31,196	0.00624	0.527	0.663	0.725	0.382	0.8789	0.00237	0.00157
14	4D	69.89	0.326	11,760	0.01524	0.404	0.814	0.629	0.254	0.7420	0.00142	0.00116
15		78.97	0.367	14,701	0.01334	0.436	0.780	0.827	0.360	0.7451	0.00162	0.00126
16		90.41	0.427	19,140	0.01101	0.473	0.734	1.053	0.499	0.7951	0.00254	0.00186
17		91.92	0.447	20,333	0.01016	0.478	0.728	1.146	0.548	0.8283	0.00260	0.00190
18		95.69	0.485	22,783	0.00892	0.490	0.712	1.024	0.502	0.8319	0.00281	0.00200
19		100.22	0.504	24,559	0.00857	0.505	0.693	1.157	0.584	0.8581	0.00347	0.00241
20		103.38	0.530	26,425	0.00796	0.514	0.680	1.361	0.700	0.8800	0.00349	0.00237
21		108.20	0.558	28,840	0.00743	0.529	0.660	1.506	0.797	0.9092	0.00455	0.00301
22		113.32	0.637	34,097	0.00590	0.545	0.638	1.889	1.029	0.9392	0.00499	0.00319
23	6D	66.21	0.324	11,166	0.01468	0.391	0.828	0.684	0.268	0.7245	0.00133	0.00110
24		78.57	0.372	14,828	0.01294	0.434	0.781	1.316	0.572	0.7765	0.00201	0.00157
25		84.97	0.406	17,314	0.01155	0.456	0.756	1.334	0.608	0.7937	0.00230	0.00174
26		91.21	0.471	21,277	0.00911	0.476	0.731	1.433	0.682	0.8239	0.00264	0.00193
27		99.16	0.528	25,499	0.00775	0.501	0.698	1.567	0.786	0.8645	0.00332	0.00232
28		103.41	0.572	28,561	0.00682	0.515	0.680	1.641	0.844	0.8826	0.00321	0.00218
29		114.11	0.629	33,849	0.00608	0.547	0.635	1.405	0.769	0.9289	0.00402	0.00255
30	8D	79.29	0.349	14,051	0.01475	0.437	0.778	0.952	0.416	0.7423	0.00208	0.00162
31		92.43	0.419	19,127	0.01164	0.480	0.726	1.108	0.532	0.8099	0.00219	0.00159
32		104.33	0.495	24,872	0.00918	0.517	0.676	1.949	1.009	0.8377	0.00325	0.00220
33		109.66	0.553	28,892	0.00763	0.534	0.654	1.958	1.045	0.9008	0.00391	0.00256
34		118.35	0.602	33,282	0.00682	0.560	0.616	1.967	1.101	0.9215	0.00422	0.00260
35		122.62	0.644	36,485	0.00613	0.573	0.598	1.594	0.913	0.9343	0.00477	0.00285
36	10D	72.16	0.313	11,619	0.01696	0.412	0.806	0.875	0.361	0.7213	0.00116	0.00094
37		75.05	0.338	12,988	0.01501	0.422	0.795	1.129	0.477	0.7240	0.00177	0.00141
38		87.40	0.402	17,514	0.01211	0.464	0.746	1.269	0.588	0.7737	0.00193	0.00144
39		94.50	0.463	21,527	0.00969	0.487	0.717	1.507	0.733	0.8072	0.00242	0.00173
40		98.53	0.528	25,371	0.00771	0.499	0.700	1.525	0.762	0.8138	0.00234	0.00164
41		102.81	0.542	26,940	0.00756	0.513	0.683	1.663	0.852	0.8549	0.00334	0.00228
42		113.56	0.637	34,151	0.00591	0.546	0.637	1.842	1.005	0.9169	0.00420	0.00267
43		119.70	0.661	36,826	0.00571	0.564	0.611	2.420	1.365	0.9284	0.00684	0.00418
44	12D	74.10	0.323	12,247	0.01634	0.419	0.798	0.531	0.222	0.6924	0.00102	0.00082
45		79.99	0.379	15,374	0.01260	0.439	0.776	0.814	0.357	0.7050	0.00110	0.00085
46		86.22	0.425	18,342	0.01067	0.460	0.751	1.135	0.522	0.7214	0.00177	0.00133
47		94.61	0.484	22,503	0.00890	0.487	0.717	1.451	0.707	0.7532	0.00218	0.00156
48		97.24	0.508	24,129	0.00826	0.495	0.706	1.539	0.762	0.7708	0.00239	0.00169
49		101.04	0.528	25,879	0.00787	0.507	0.690	1.546	0.784	0.7893	0.00266	0.00184
50		107.28	0.558	28,652	0.00738	0.527	0.664	1.589	0.837	0.8228	0.00290	0.00192
51		109.66	0.612	31,943	0.00624	0.534	0.654	1.613	0.861	0.8458	0.00421	0.00275
52		118.33	0.623	34,420	0.00637	0.560	0.616	1.676	0.938	0.8965	0.00480	0.00296
53		124.15	0.634	36,269	0.00637	0.577	0.591	2.048	1.182	0.9277	0.00545	0.00322
54	16D	70.62	0.308	11,233	0.01716	0.407	0.812	0.724	0.295	0.7205	0.00106	0.00086
55		77.18	0.346	13,611	0.01468	0.430	0.787	0.941	0.404	0.7325	0.00190	0.00150
56		85.51	0.414	17,743	0.01117	0.457	0.754	1.041	0.476	0.7751	0.00226	0.00170
57		89.63	0.459	20,413	0.00948	0.471	0.737	1.163	0.548	0.7898	0.00230	0.00169
58		94.57	0.500	23,231	0.00834	0.487	0.717	1.284	0.625	0.8177	0.00268	0.00192
59		101.96	0.538	26,531	0.00765	0.510	0.686	1.341	0.684	0.8579	0.00340	0.00233
60		106.63	0.588	30,054	0.00661	0.524	0.666	1.399	0.734	0.8861	0.00384	0.00256
61		111.80	0.623	32,999	0.00611	0.540	0.644	1.443	0.780	0.9053	0.00407	0.00263
